# Longitudinal Changes of Depressive Symptoms in Sedentary Women Who Exercised During Pregnancy

**DOI:** 10.1089/whr.2023.0028

**Published:** 2023-10-31

**Authors:** Lauren E. Hicks, SeonAe Yeo

**Affiliations:** School of Nursing, University of North Carolina at Chapel Hill, Chapel Hill, North Carolina, USA.

**Keywords:** depression, depressive symptoms, pregnancy, postpartum period, mood disorders

## Abstract

**Introduction:**

Prenatal depression is a common disorder; however, little is known about how depressive symptoms manifest during pregnancy, including when symptoms present and what symptoms are common. This study aimed to better understand prenatal depressive symptoms during pregnancy in the postpartum period, as well as how exercise, such as walking and stretching, can improve depressive symptoms during pregnancy and the postpartum period.

**Methods:**

A total of 55 women were assessed using the Beck Depression Inventory-II for depressive symptoms at 16 weeks, 28 weeks, and 2 months postpartum. Sedentary pregnant women at-risk for preeclampsia were randomly assigned to either a stretching or walking group for 40 minutes five times a week from 18 weeks of gestation until birth. The primary analyses were analysis of variance and mixed-effects models.

**Results:**

All depressive symptoms decreased throughout pregnancy during the postpartum period, although this trend was not statistically significant. Cognitive-affective and somatic depressive symptoms had different trajectories during pregnancy into the postpartum period, but no significant difference was found. Statistically significant improvements were observed in loss of energy and change in sleeping pattern for the walking and stretching groups.

**Conclusion:**

The finding that physical activity improves the depressive symptoms' loss of energy and changes in sleeping patterns during pregnancy aligns with the existing literature, but little research has examined how individual depressive symptoms change throughout pregnancy into the postpartum period. Gaining a better understanding of the trajectories and manifestations of depressive symptoms during pregnancy and the postpartum period is essential for improving detection and treatment practices. Understanding when and how depressive symptoms are present is critical for the clinical diagnosis of this disorder.

## Introduction

Depression is the most common perinatal disorder, with 18% of pregnant women experiencing symptoms, including guilt, constant worry, obsessive thoughts, extreme sadness, fatigue, anxiety attacks, and difficulty carrying out daily tasks.^1^ Serious maternal mental illness, such as depression, is independently associated with an increased risk of adverse birth outcomes, such as preeclampsia and preterm birth.^2^ If left untreated, prenatal depression can lead to postpartum or major depressive disorder, anxiety disorders, and severe maternal morbidity.^1,3^

Aspects of prenatal depression that lack sufficient understanding include the timing that depressive symptoms present and type of depressive symptoms experienced by pregnant women. Interventions to reduce prenatal depression risk, such as exercise and stress reduction, must be better understood. In addition, there is no consensus on the trajectory of depressive symptoms during pregnancy. An increased knowledge of depressive symptoms during pregnancy is essential for assessing and diagnosing this disorder. Currently, a common obstetric practice is to screen for prenatal depression once during the perinatal period, including the antepartum and postpartum periods.^4^

Although the timing of the presentation of depressive symptoms during pregnancy is necessary to improve detection, the types of depressive symptoms commonly observed during pregnancy need to be thoroughly explored. Depressive symptoms include somatic and cognitive and affective symptoms.^5^ Cognitive and affective depressive symptoms are commonly categorized together and include symptoms such as negative mood or irritability.^5^ Somatic depressive symptoms are closely related to bodily awareness and include symptoms such as loss of energy and fatigue.^5^ Many symptoms used to assess prenatal depression are identical or are similar to those of pregnancy, complicating the diagnosis of prenatal depression.

An in-depth understanding of how prenatal depression manifests during pregnancy is needed with risk factors and risk-reduction interventions requiring improved insight. An accessible and effective risk-reducing intervention is physical activity, such as walking and stretching, which has been found to decrease the risk of preeclampsia, gestational hypertension, gestational diabetes, weight gain, and postpartum depression.^6^ Physical activity known to be safe during pregnancy, including moderate-intensity aerobic activity, muscle-strengthening, and gentle stretching, and is not associated with an increased risk of miscarriage, stillbirth, or delivery complications, as previously believed.^6^

Unfortunately, the impact of physical activity has not been thoroughly explored for prenatal depression. In one systematic review of exercise during pregnancy and prenatal depression, Sánchez-Polán et al. found that supervised exercise during pregnancy may be useful for the prevention and reduction of prenatal depression and depressive symptoms.^6^ In addition, inactive women were at a 16% higher risk of developing prenatal depression than mildly to moderately active women.^7^ While these findings are promising, a more comprehensive understanding is needed.

This study aimed to better understand prenatal depressive symptoms during pregnancy in the postpartum period as well as how walking and stretching influence depressive symptoms during pregnancy and the postpartum period. Addressing this issue is important to improve the detection and treatment of prenatal depression. The study's research questions were (1) to determine whether depressive symptoms decrease throughout pregnancy into the postpartum period, (2) to determine whether there were differences in somatic and cognitive-affective depressive score trajectories during pregnancy and the postpartum period, and (3) to determine whether walking or stretching reduces the risk of developing or worsening depressive symptoms during pregnancy.

## Materials and Methods

A total of 55 women were assessed twice during pregnancy at 16 weeks' gestation (WG), 28 WG, and at 2 months postpartum (MPP). The design and methods of the study have been published elsewhere.^8^ Sedentary pregnant women at-risk for preeclampsia were randomly assigned to either a stretching or walking group.

### Participants

One-hundred thirty-seven women were recruited before 14 WG, between January 2002 and August 2006. Participants were randomized to a walking or stretching group and exercised for 40 minutes 5 days a week from 18 WG until birth.^8^ Of the 137 participants, 81 were randomized to the walking group and 58 to the stretching group. Eligible participants were women who had been diagnosed with preeclampsia during a previous pregnancy, had lower than average cardiovascular fitness, or had a sedentary lifestyle.

### Methods and study procedure

Of the 137 participants, 55 answered the Beck Depression Inventory II (BDI-II), which we report here.^9^ The Beck Depression Inventory is a 21-item tool that uses a multiple-choice format to measure the presence and degree of depression consistent with the Diagnostic and Statistical Manual of Mental Disorders, Fifth Ediction.^9^ Scoring for the BDI-II considers scores of 0–9 as no or minimal depression, 10–18 as mild-to-moderate depression, 19–29 as moderate-to-severe depression, and 30–63 as severe depression.^9^ The BDI-II has been tested for reliability and validity, determining that the BDI-II is capable of discriminating between groups that contrasted in the level of depression and has an internal consistency with a correlation coefficient of .89.^10^ The BDI-II was used to assess depressive symptoms at 16 WG, 28 WG, and 2 MPP.

### Statistical data

All statistical analyses were performed using SPSS version 28 (IBM Corp., Armonk, NY). Descriptive statistics were performed for age in years, number of pregnancies including the current one, ethnicity, education, and number of perceived support persons. Descriptive statistics were also performed to evaluate the prevalence of depressive symptoms at 16 WG, 28 WG, and 2 MPP and to compare the prevalence of cognitive-affective and somatic depressive symptoms at 16 WG, 28 WG, and 2 MPP.

### Statistical analysis

The same subjects completed the BDI-II at 16 WG, 28 WG, and 2 MPP; therefore, the primary analyses conducted were analysis of variance (ANOVA) and mixed-effects models, with a 95% CI. The ANOVA involved repeatedly and randomly permuting the collected data among groups, calculating the test statistics, and comparing the distribution under the null hypothesis.^11^ The mixed-effects models allowed for within-subject correlation of repeated measurements and between-subject variation.^12^

## Results

Demographic information was collected from the participants (Table 1). No statistically significant difference was found in the BDI-II scores, which decreased throughout pregnancy into the postpartum period. Nonsignificant trends were found among cognitive-affective and somatic BDI-II item score trajectories at 16 WG, 28 WG, and 2 MPP. Walking and stretching were found to reduce different depressive symptoms at 28 WG and 2 MPP.

**Table 1.** Demographic Information Frequencies and Percentages of 55 Pregnant Women at Increased Risk for Preeclampsia

Variables	Value, ***n*** (%)
Age (years)	
20–29	13 (23.6)
30–39	39 (70.9)
40–42	3 (5.5)
Number of pregnancies	
1	24 (43.6)
2	14 (25.5)
3	10 (18.2)
4	6 (10.9)
≥5	1 (1.8)
Ethnicity	
Black/African American	3 (5.5)
Asian or Pacific Islander	2 (3.6)
Hispanic/Latina	1 (1.8)
White/non-Hispanic	44 (80.0)
Multiracial	2 (3.6)
Other	3 (5.5)
Education	
<High school diploma	1 (1.8)
High school diploma	10 (18.2)
Some college	13 (23.6)
Bachelor's degree	19 (34.5)
Master's degree	7 (12.7)
Doctoral degree	5 (9.1)
Support persons	
0	4 (7.5)
1	9 (16.4)
2	5 (9.1)
3	8 (14.5)
4–10	19 (34.6)
≥11	8 (14.5)

**FIG. 1.**
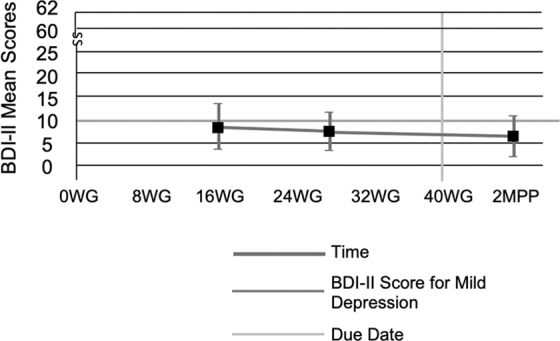
BDI-II mean score at 16 and 28 WG, and 2 MPP. BDI-II, Beck Depression Inventory II; MPP, months postpartum; WG, weeks' gestation.

The first research question was tested to determine whether depressive symptoms decreased throughout pregnancy and in the postpartum period. Fifty-five pregnant sedentary women were assessed for depressive symptoms using the BDI-II at 16 WG, 28 WG, and 2 MPP. In total, 135 scores were obtained. Although the BDI-II mean scores decreased throughout pregnancy into the postpartum period, the trend was not significant (Fig. 1).

**FIG. 2.**
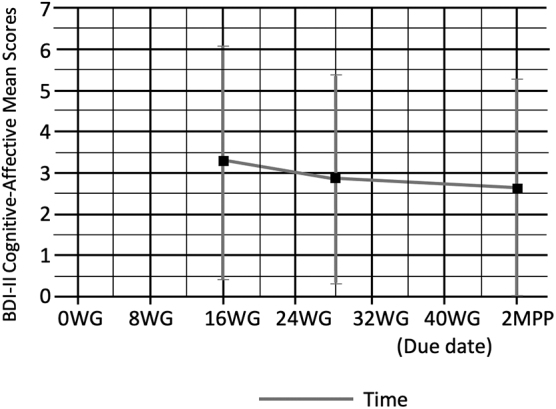
BDI-II cognitive-affective mean scores at 16 and 28 WG, and 2 MPP. BDI-II, Beck Depression Inventory II; MPP, months postpartum; WG, weeks' gestation.

Next, we determined whether there were differences in somatic and cognitive-affective depressive score trajectories during pregnancy and the postpartum period. A nonsignificant difference was found among 55 sedentary pregnant women between cognitive-affective (*F*(2,132) = 0.699, *p* = 0.51) and somatic (*F*(2,132) = 1.58, *p* = 0.21) BDI-II mean score trajectories. Cognitive-affective symptoms were the most prevalent at 16 WG (mean [*M*] = 3.28, standard deviation [SD] = 2.90), followed by a decrease at 28 WG (*M* = 2.85, SD = 2.58), and symptoms decreased slightly more at 2 MPP (*M* = 2.62, SD = 2.12) (Fig. 2).

**FIG. 3.**
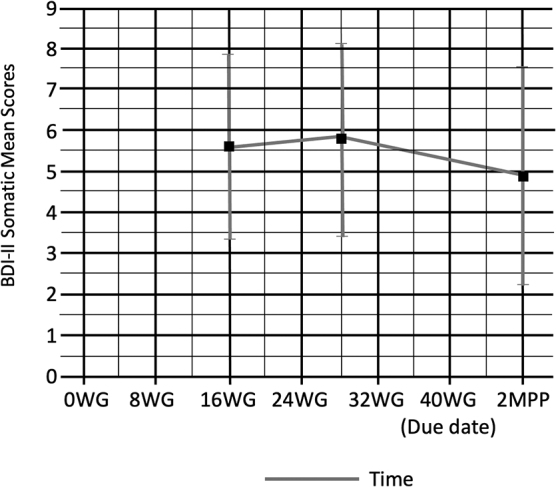
BDI-II somatic mean scores at 16 and 28 WG, and 2 MPP. BDI-II, Beck Depression Inventory II; MPP, months postpartum; WG, weeks' gestation.

At 16 WG, somatic symptoms were prevalent when compared with cognitive-affective symptoms (*M* = 5.57, SD = 2.27), then increased slightly at 28 WG (*M* = 5.72, SD = 2.33) and decreased at 2 MPP (*M* = 4.86, SD = 2.68) (Fig. 3). Cognitive-affective and somatic items scores had differing trajectories throughout pregnancy into the postpartum period, but the difference was not significant.

**FIG. 4.**
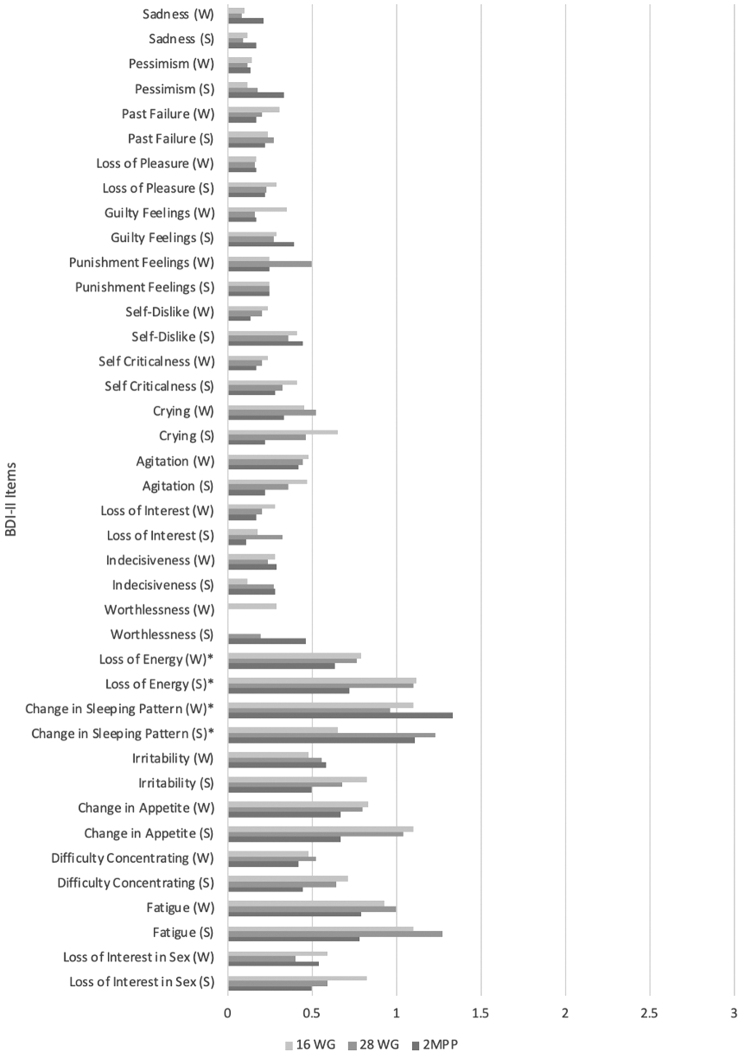
BDI-II mean scores per item between the walking and stretching groups at 16 and 28 WG, and 2 MPP. *p < .05. BDI-II, Beck Depression Inventory II; MPP, Months postpartum; S, stretching group; W, walking group; WG, weeks' gestation.

Finally, we determined whether types of exercise, walking and stretching, affect symptom relief differently. Fifty-five pregnant sedentary women randomly assigned to walking or stretching groups were assessed for depressive symptoms using the BDI-II at 16 WG, 28 WG, and 2 MPP. During the three assessments, 31 BDI-II assessments were collected from the walking group and 23 from the stretching group. Significant differences between the walking and stretching groups were found in somatic depressive items, including loss of energy (*p* = 0.002) and changes in sleeping pattern (*p* = 0.026) (Fig. 4). Walking significantly improved the loss of energy at 28 WG (*p* = 0.01) and 2 MPP (*p* < 0.001), but was not significant at 16 WG. Walking also significantly improved changes in sleeping pattern at 2 MPP (*p* = 0.001), but not at 16 WG or 28 WG.

Stretching significantly improved the loss of energy at 2 MPP (*p* = 0.006), but not at 16 WG or 28 WG. We also find stretching significantly improved changes in sleeping pattern at 28 WG (*p* = 0.01) and 2 MPP (*p* = 0.03), but not at 16 WG. No significant differences were found among the other items in the BDI-II.

## Discussion

Although no significant trajectory of depressive symptoms has been found, depressive symptoms decreased throughout pregnancy into the postpartum period. Cognitive-affective and somatic depressive symptoms had different trajectories during pregnancy into the postpartum period, but no significant difference was found. In addition, light-to-moderate exercises, such as stretching or walking, have been found to improve the symptoms of loss of energy or change in sleeping patterns during pregnancy.

The first research question, whether depressive symptoms decrease throughout pregnancy into the postpartum period, did and did not align with existing findings due to the inconsistencies of findings in previous research studies. For example, Truijens et al. found different patterns of depressive symptoms during pregnancy, with the lowest scores occurring during the first trimester and highest rates in the second trimester, followed by a slight decrease in symptoms in the third trimester.^13^ Our study findings aligned with these findings. Our study first assessed for depression in the second trimester, which had the highest scores, followed by a decrease in scores in the third trimester, which continued into the postpartum period.

Another study by Frederiksen et al. used data from 1036 women from midpregnancy to 12 MPP and found four classes of depressive symptom trajectories, including symptoms only during pregnancy, symptoms only during postpartum, symptoms being moderate and persistent throughout pregnancy and postpartum, and minimal symptoms throughout pregnancy and postpartum.^14^ Our analysis observed depressive symptoms being minimal or moderate and persisting throughout pregnancy into the postpartum period.^10^ Our findings do not align with all four trajectories, but do suggest that depressive symptoms fluctuate during pregnancy into the postpartum period.

Our findings did not align with Kolomańska et al. who found that depressive symptoms are high in the first trimester due to increases in various hormones and fear of miscarriage, while in the second trimester, emotions stabilize, but in the third trimester, there is a renewed increase in anxiety and uncertainty.^15^ Our study contradicts these findings, with depressive symptoms being highest in the second trimester and gradually decreasing throughout pregnancy into the postpartum period.

Existing literature on the trajectory of depressive symptoms during pregnancy and the postpartum period is inconsistent, evident by our findings aligning with some studies, but not all. The lack of knowledge about depressive symptoms during pregnancy leads to inconsistent education of obstetric providers, which interferes with their ability to detect and diagnose perinatal depression. The lack of consensus of depressive symptom trajectories during pregnancy can make detecting the disorder more difficult. Based on the study findings, the reason depressive symptoms increased throughout pregnancy and decreased during the postpartum period is unknown. Additional research is needed to detect the underlying cause.

The second research question, to determine whether there is a difference in somatic and cognitive-affective depressive symptoms during pregnancy and the postpartum period, was not statistically significant. Few studies have examined the differences between somatic and cognitive-affective depressive symptoms, and no study has explored these differences among pregnant women, and therefore, our findings could not be compared with other research findings. There is a possibility that a statistically significant difference exists for some pregnancy women, but based on our sample, we were unable to detect a significant difference.

To determine whether walking or stretching reduces the risk of developing or worsening depressive symptoms during pregnancy, the third research question identified two depressive items that were improved by walking and stretching. Several studies have explored the effects of different exercise types on depressive symptoms during pregnancy. El-Rafie et al. found that regular exercise, including walking, aerobic exercise, stretching, and relaxation, significantly improves depressive symptoms among pregnant women.^16^ These findings align with our research, but do not specify the effects of different forms of exercise or the specific depressive symptoms that were influenced by different forms of exercise.

Two systematic reviews were found to look at exercise and depressive symptoms during pregnancy. A systematic review by Kolomańska et al. found that, based on 17 articles, physical activity at least once a week significantly reduces depressive symptoms.^15^ Another systematic review by Davenport et al. examined 52 articles and found that when pregnant women participated in exercise for at least 644 minutes/week, which is 90 minutes per day, the risk and severity of prenatal depression are reduced.^17^ While both reviews found that exercise improves depressive symptoms, the efficacy of different types of exercise is not specified and the amount of time needed to improve symptoms is large, ranging from once a week to 90 minutes per day.

The current lack of understanding of how types of exercise and duration of exercise improve depressive symptoms inhibits pregnant women from using exercise effectively to manage or improve their depressive symptoms during pregnancy. In our study, walking and stretching were found to improve loss of energy throughout pregnancy into the postpartum period. The underlying reason is unknown and further research is needed.

The finding that physical activity improves depressive symptoms during pregnancy aligns with the existing literature; however, no research has examined how individual depressive symptoms change throughout pregnancy and in the postpartum period. A better understanding of depressive symptoms and the impact of walking and stretching on them can provide guidelines for obstetric providers to educate pregnant patients. Better knowledge of prenatal depressive symptoms can also provide pregnant women more accurate information to form realistic expectations of thoughts and feelings during pregnancy, as well as if depressive symptoms are normal or abnormal.

The novelty of these results calls for various research opportunities. Perhaps the most important is gaining a better understanding of the patterns and manifestations of depressive symptoms during pregnancy and in the postpartum period to improve detection and intervention practices. Understanding when and how depressive symptoms are present is critical for clinical diagnosis of this disorder.

A possible limitation of this study is that the population was exclusively sedentary pregnant women who were at high risk of developing preeclampsia. Future research should explore the trajectories of somatic and cognitive-affective depressive symptoms among women with low-risk pregnancies or different types of high-risk pregnancies.

Future exploration of the effects of different types of exercise, such as walking and stretching, on prenatal depressive symptoms could aid in treating this disorder. Further research on the effects of a light-to-moderate walking program during pregnancy would be beneficial for understanding how walking can improve depressive symptoms. Regular stretching has been found to ease the loss of energy and changes in sleeping patterns during pregnancy in sedentary women. Further research exploring how regular stretching affects depressive symptoms could help improve prenatal depression.

## Conclusions

Prenatal depression is a common and complex disorder that can lead to negative birth outcomes in women and infants.^1^ To improve the detection and treatment of this disorder, a comprehensive understanding of the manifestation of depressive symptoms, such as timing during pregnancy and type, and risk reduction interventions, such as walking or stretching, is needed. The results provide evidence for the need to increase understanding of prenatal depression trajectories, the prevalence of differing depressive symptoms, and how exercise such as walking and stretching can improve depressive symptoms, specifically loss of energy and changes in sleeping patterns. Further research is needed to increase scientific knowledge on prenatal depression.

## Data Access Statement

The research data supporting this document were obtained from S.Y. and are available on request.

## Abbreviations Used

ANOVA: analysis of variance
BDI-II: Beck Depression Inventory-II
*M*: mean
MPP: months postpartum
NINR: National Institute of Nursing Research
SD: standard deviation
WG: weeks' gestation

## References

[1] National Institute of Health. What factors increase the risk of maternal morbidity and mortality? Available from: https://www.nichd.nih.gov/health/topics/maternal-morbidity-mortality/conditioninfo/factors [Last accessed: September 6, 2022].

[2] Heun-Johnson H, Seabury, SA, Menchine M, et al. Association between maternal serious mental illness and adverse birth outcomes. J Perinatol 2019;39:737–745; doi: 10.1038/s41372-019-0346-5 30850757 PMC6503973

[3] National Institute of Nursing Research. Recurrence of severe maternal morbidity in second pregnancy. Available from: https://ninr.nih.gov/researchandfunding/researchhighlights/recurrence-of-severe-maternal-morbidity [Last accessed: August 31, 2022].

[4] American College of Obstetricians and Gynecologists. Screening for perinatal depression. Available from: https://www.acog.org/clinical/clinical-guidance/committee-opinion/articles/2018/11/screening-for-perinatal-depression [Last accessed: November 15, 2022].

[5] Beck A, Steer R, Brown G. Beck Depression Inventory Manual, 2nd edition. The Psychological Corporation: San Antonio, TX, USA.

[6] World Health Organization. WHO Guidelines on physical activity and sedentary behavior; 2020; pp. 47–51. Available from: https://www.who.int/publications-detail-redirect/9789240015128 [Last accessed: May 8, 2023].

[7] Sánchez-Polán M, Franco E, Silva-Jośe C, et al. Exercise during pregnancy and prenatal depression: A systematic review and meta-analysis. Front Physiol 2021;12; doi: 10.3389/fphys.2021.640024 PMC8273431 34262468

[8] Yeo S. A randomized comparative trial of the efficacy and safety of exercise during pregnancy: Design and methods. Contemp Clin Trials 2006;27:531–540. 16861054 10.1016/j.cct.2006.06.005

[9] Beck AT, Ward CH, Mendelson M, et al. An inventory for measuring depression. Arch Gen Psychiatry 1961;4:561–571; doi: 10.1001/archpsyc.1961.01710120031004 13688369

[10] Sacco R, Santangelo G, Stamenova S, et al. Psychometric properties and validity of Beck Depression Inventory II in multiple sclerosis. Eur J Neurol 2016;23:744–750; doi: 10.1111/ene.12932 26782789

[11] Sthle L, Wold S. Analysis of variance (ANOVA). Chemom Intell Lab Syst 1989;6:259–272; doi: 10.1016/0169-7439(89)80095-4

[12] Brysbaert M, Stevens M. Power analysis and effect size in mixed effects models: A tutorial. J Cogn 2018;1:9; doi: 10.5334/joc.10 31517183 PMC6646942

[13] Truijens SEM, Spek V, van Son MJM, et al. Different patterns of depressive symptoms during pregnancy. Arch Womens Ment Health 2017;20:539–546; doi: 10.1007/s00737-017-0738-5 28593361 PMC5509781

[14] Frederiksen E, von Soest T, Smith L, et al. Patterns of pregnancy and postpartum depressive symptoms: Latent class trajectories and predictors. J Abnorm Psychol 2017;126:173–183; doi: 10.1037/abn0000246 27935730

[15] Kolomańska D, Zarawski M, Mazur-Bialy A. Physical activity and depressive disorders in pregnant women—A systematic review. Medicina 2019;55:212; doi: 10.3390/medicina55050212 31130705 PMC6572339

[16] El-Rafie MM, Khafagy GM, Gamal MG. Effect of aerobic exercise during pregnancy on antenatal depression. Int J Womens Health 2016;8:53–57; doi: 10.2147/IJWH.S94112 26955293 PMC4772941

[17] Davenport MH, McCurdy AP, Mottola MF, et al. Impact of prenatal exercise on both prenatal and postnatal anxiety and depressive symptoms: A systematic review and meta-analysis. Br J Sports Med 2018;52; doi: 10.1136/bjsports-2018-099697 30337464

